# EBV in pediatric neoplasia – intensity of infection as independent prognostic factor

**Published:** 2012-09-25

**Authors:** S Căinap, A Răchisan, B Fetică, R Cosnarovici, E Mihut, G Popa, D Gheban, C Căinap

**Affiliations:** * ”Iuliu Hatieganu” University of Medicine and Pharmacy, Cluj Napoca; ** Pediatric Emergency County Hospital, Cluj Napoca; *** “I. Chiricută” National Institute of Oncology, Cluj Napoca

**Keywords:** Epstein Barr virus, malignancy, prognostic factor

## Abstract

**Rationale: **Cancer disease is continuously rising worldwide as far as its incidence is concerned. Efforts were made in order to identify the etiologic factors. A good example for exogenous factors is Epstein Barr virus (EBV) which is largely spread worldwide, over 90% of the adult general population being infected by it. EBV is believed to be implicated in Burkitt lymphoma, Hodgkin lymphoma, nasopharyngeal carcinoma, etc.

**Objective: **In this paper, we will try to present the experience of two centers in Cluj County involved in the treatment of pediatric cancer, focusing on the influence of the presence of Epstein Barr virus in the outcome of the neoplasia.

**Methods and Results:** we took into account the clinical data regarding histology, stage of the disease, titer of specific antibodies for EBV, serological and imagistic evaluations of the patients treated in a retrospective consecutive manner for 5 years - 2005-2010.

Regarding our cohort of 120 patients, we analyzed the items in the paper in detail together with the statistical analysis and searched for a link between the intensity of the infection of EBV and response, disease, free survival, toxicities of the treatment.

**Discussion:** there are few data concerning the influence of EBV regarding the outcome of pediatric neoplasia. The published studies suggest a positive influence of EBV especially in Hodgkin disease mixed cellular subtype. In this study, EBV negative patients do better than the EBV positive, but the infection with EBV protects the patients against hematological toxicities.

## Introduction

The incidence of cancer is in a continuous elevation, the latest data suggest that the “chance” of a neoplasm in male is of 50% and in female it is of about 30% [**1**]. Intensive efforts were made in order to identify the etiology of cancer but until now, we only know that multiple factors are implicated in cancerogenesis. For the time being, it is certified that even an infectious agent could initiate a neoplasia. Extensive literature is debating the role of Epstein Barr virus (EBV) in this disease. EBV is a virus in Herpesviridae family identified in 1964 in a Burkitt cell line [**2**] and after many years of research WHO declared EBV as the first class of carcinogen [**3**].

## Aim of the study

In a previously published series of patients with neoplasia and infection with EBV, we identified a cut off value of IgG VCA with a prognostic value concerning the probability of response to chemotherapy. In this study, we performed a more extensive analysis with a larger population.

## Patients and methods

We selected patients treated in a retrospective and consecutive manner in the National Institute of Oncology Cluj Napoca and Department of Oncology of Pediatric Emergency County Hospital Cluj between 2005 and 2010. Our cohorts are made up of 120 patients with malignant Hodgkin or non-Hodgkin lymphoma or other solid tumor certified with Epstein Barr by the positivity of IgG VCA antibody. We took into account clinical data from their clinical files, including patient demographics, hematology, erythrocyte sedimentation rate, albumin, lactic dehydrogenase level, clinical disease stage, positivity of B signs, bulky disease (> 10 cm or mediastinal mass more than one third of thoracic diameter) and we calculated the International Prognostic Scores (IPI), type of treatment, response to treatment. All the histologies were reviewed in order to assure a consistent diagnosis of the patients with clinical files. Inclusion criteria were: histological proved with malignant lymphoma, age between 1-18 years, treated by standard chemotherapy, EBV infection confirmed by positivity of IgG anti VCA. We excluded the patients unable to finish the chemotherapy or with incomplete clinical data or EBV negative. In our study, we had 38 girls and 82 boys with an evidence prevalence of males with a statistical significance (p=0,031) but not related to IgG VCA titer. The median age was of 11,16 respectively 11,26 years, the difference being insignificant. 

As previously mentioned, we have identified a cut off value of IgG VCA = 213 UI/ml by a previous statistical analysis, which could be an independent prognostic factor in pediatric neoplasia. We used the statistical procedures indicated for the aim of this paper, descriptive or inferential techniques. Chi square or Fisher Exact Test was used to evaluate the correlations between quantitative variables, following standard application criteria for each test. Normality of continuous data was tested by using Shapiro-Wilk test. Based on normality testing results, between-group differences in continuous data were assessed using either Student T test or Mann and Whitney U Test. R.O.C. analysis, including: curve construction, area under curve (AUC) with 95% confidence interval (95% CI) and cut-off determination was performed. Sensitivity and Specificity were also determined for the identified cut-off value. A significance threshold of p<=0.05 was selected for all tests. SPSS 13.0(Chicago, Il) and MedCalc 8.3.1.1 software applications were used for data analysis.

The main patient’s characteristics are listed in the following table.

**Table 1:** The main patient’s characteristics

Patients	IgG VCA < 213,4	IgG VCA > 213,4	p value
Age
Mean	11,16	11,22	0,911
Sex
male	26	34	0,031
female	12	17	
Histological types
Hodgkin lymphoma	10	28	0,002
Non-Hodgkin lymphoma	21	12	0,414
Other solid tumor	20	29	2,02
TNM stage or Ann Arbor
Stage I and II	13	16	
Stage III and IV	21	26	1,006
Extra nodal involved sites
Bowel	4	8	
Spleen	1	2	
Liver	1	4	
Other	4	10	
LDH values
Elevated – mean	599,31	727,59	0,89
Prognostic group
High risk	9	11	
Intermediary risk	8	13	0,278
Low risk	6	10	
IPI score
Mean	2,38	2,41	0,884
Performance status
Mean	2,41	2	0,017
Disease free survival	32 pts	46 pts	
events	1	7	0,083

If we look at the different subtypes of lymphoma in our population, there is no statistical correlation between histology and intensity of EBV infection and no link between EBV and histological subtype, as it can be seen in **Table 2**

**Table 2:** Correlations between histological subtype and titer of IgG VCA

Patients Histological subtypes NHL	IgG VCA < 213,4	IgG VCA > 213,4	p value
Burkitt	9	8	
Large B cell	3	4	0,357
T cell	2	0	
Histological subtypes HL
Nodular sclerosis	8	11	
Mixed cellular	3	5	0,971
Lymphoid depletion	3	4	

Other viral coinfection was searched in our population: for hepatic virus C we had 10% coinfection (7 pts. / 70) and 17, 8 % for B virus (13 / 73 pts.). Cytomegalovirus was present in 10/ 70 pts. 14, 28%. As shown in **Table 3** none of these coinfections has a statistical significance regarding IgG VCA titer.

**Table 3:** Viral coinfection

Patients Virus C hepatic	IgG VCA < 213,4	IgG VCA > 213,4	p value
absent	24	40
present	3	4	0,688
Virus B hepatic
absent	23	37
present	6	7	0,601
Cytomegalovirus
absent	23	37
present	4	6	1,000

Concerning the toxicities during chemotherapy in the previous study report we did not find any influence of the presence of EBV, but we were aware that the number of patients (the first 35 patients were included in the present study) was not enough for definitive conclusions. We reanalyzed the final populations and we only mentioned the item and cycles where it reaches statistical significance in **Table 4**.

**Table 4:** Hematological toxicities during the treatment correlate with titer of IgG VCA

Patients Toxicities – platelets (median value – nadir)	IgG VCA < 213,4 38 pts	IgG VCA > 213,4 50 pts	p value
Cycle 1 – initial value	475	329	0,011
Cycle 3 – nadir	131	224	0,03
Cycle 4 – nadir	120	263	0,000
Hemoglobin (median value – nadir)	24 pts	49 pts	
Cycle 2	9,7	11,65	0,002
Cycle 3	9,4	11	0,007
Cycle 4	9,55	11,5	0,003
Neutrophils (median value)	37 pts	48 pts	
Cycle 1	6,9	4,73	0,023
Cycle 2	4,18	2,70	0,009
Lymphocytes (median value – nadir)	22 pts	21 pts	
Cycle 2	0,58	1,3	
Cycle 3	0,7	1,06	0,02
Cycle 4	0,55	1,33	0,003
Monocytes (median value – nadir)	37 pts	47 pts
Cycle 2	0,04	0,3	0,008
Cycle 3	0,16	0,48	0,001
Cycle 4	0,04	0,36	0,006

**Fig. 1:**
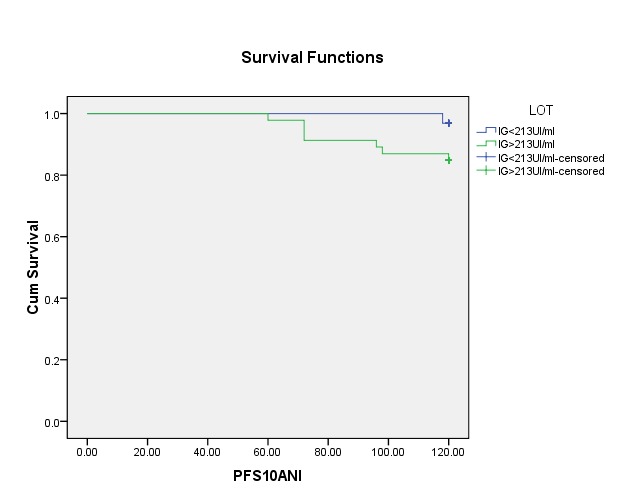
Progression free survival in correlation with IgG VCA titer

Finally, we studied if the intensity of EBV infection has a role in the outcome of pediatric neoplasia. For that, we compared the disease free survival (DFS) period (counted from first chemotherapy administration until first date of relapse) between 2 subgroups: IgG VCA < 213 UI/ml and > 213 UI/ml. The result is negative – the 2 curves are similar as it can be seen in **Figure 1**.

## Discussion

The presence of EBV in patients with HD or non-HD is variable. The published studies showed that it is a geographic variation: Asian population appear to have a low frequency of HD with intermediary level of EBV [**4**]. 

The role of EBV in the outcome of pediatric neoplasia is not very well known. The present paper has tried to offer a general view in one well-known oncologic center. The incidence of intense EBV infection (interpreted as high level of IgG VCA – more than 213 UI/ml – cut off value identified in a previous study) is of approximately 57,30% (51/89 patients included). The rate of intense infection with EBV seen in this study is similar with the one in the less developed countries [**6**], the western countries, and it is characterized by a low incidence of EBV infection [**5,7,8**].

In the previously published study, no difference was seen between groups with IgG VCA < or > 213 UI/ml for disease free survival, confirmed by the present results. The difference is regarding the statistical link EBV IgG VCA and the chance of complete response at chemotherapy – significant in first paper – unconfirmed by present study with more patients.

What is the role of EBV infection in the outcome of pediatric neoplasia? We have discordant data concerning its role (if any). Montalban [**8**] suggested a positive role with better overall survival and higher complete response in Hodgkin disease. Kwon [**9,10**] supported these findings. On the contrary, Jarrett [**11**] found a statistical significant better outcome for the EBV negative patient. Claviez [**12**] underlined that with modern chemotherapy the latent infection with EBV has no role in the general prognostic of neoplasia. However, EBV plays a role not only in the hematological neoplasia. Twu [**13**] found a more frequent relapse of those with EBV positive, in nasopharyngeal cancer. Despite the fact that we did not highlight a survival advantage, the DFS curve showed a tendency of better survival for EBV negative (marginally significance p=0,08).

The presence of EBV is not associated with clinical presentation at diagnosis, TNM stage, LDH, histology, subgroups, extra nodal involvement, prognostic group, IPI score and viral coinfection (**Tables 1,2,3**).

The presence of EBV did not emphasize the hematological toxicities secondary to the chemotherapy regimen in our previous study. On the contrary, EBV with intense infection seems to be a protective factor for increased toxicities for neutrophils, thrombocytes, lymphocytes, monocytes and hemoglobin in the actual study. We have published data from Ishii [**14**] that showed the role of monocytes, which have a positive interaction with lymphoma and contribute to lymphoma progression. The increased level of monocytes during treatment in subgroup with IgG VCA> 213 UI/ml could explain why these patients finally have a diminished disease free survival. Even in brain lymphoma, Utsuki [**15**] had posted that EBV negative patients have better general outcome.

Another interesting fact is that doxorubicin, which is an essential drug for standard chemotherapy in pediatric and adult hematological chemotherapy, has been proved to reactivate more the EBV infection; more than other chemotherapy drug such as vepeside, a reactivation which is serologically seen in our study, by titer of IgG VCA [**16**]. Machado [**17**] observed that the decrease of EBV viral load was associated with an improved therapy response at chemotherapy. 

In conclusion, our study revealed the interesting fact that the intense infection with EBV (high titer of IgG VCA) seems to be related with a decreased disease free survival. Boys seem to be more often affected regardless of the intensity of EBV infection. No histological subtype, stage, IPI score or LDH level linked statistically to the EBV exposure. A patient with intensive infection of EBV seems to have less toxicity during chemotherapy, but no difference regarding the chance of complete response.

## References

[1] Murakami  M, Robertson  ES (2007). Molecular biology of EBV in relationship to AIDS-associated oncogenesis. Cancer Treat Res.

[2] Epstein  MA, Achong  BG, Barr  YM (1964). Virus particles in cultured lymphoblasts from Burkitt's lymphoma. Lanceti.

[3] Chang  MS, Kim  WH (2005). Epstein-Barr virus in human malignancy: a special reference to Epstein-Barr virus associated gastric carcinoma. Cancer Res Treat.

[4] Cartwright  RA, Watkins  G (2004). Epidemiology of Hodgkin’s disease: a review. Hematol Oncol.

[5] Clarke  CA, Glaser  SL, Dorfman  RF (2001). Epstein-Barr virus and survival after Hodgkin’s disease in a population-based series of women. Cancer.

[6] Glaser  SL, Lin  RJ, Stewart  SL (1997). Epstein-Barr virus–associated Hodgkin’s disease. Epidemiologic characteristics in international data. Int J Cancer.

[7] Engel  M, Essop  MF, Close  P (2000). Improved prognosis of Epstein-Barr virus associated childhood Hodgkin’s lymphoma: study of 47 South African cases. J Clin Pathol.

[8] Montalban  C, Abraira  V, Morente  M (2000). Epstein-Barr virus latent membrane protein 1 expression has a favorable influence in the outcome of patients with Hodgkin’s disease treated with chemotherapy. Leuk Lymphoma.

[9] Kwon  JM, Park  YH, Kang  JH (2006). The effect of Epstein-Barr virus status on clinical outcome in Hodgkin’s lymphoma. Ann Hematol.

[10] Herling  M, Rassidakis  GZ, Vassilakopoulos  TP (2006). Impact of LMP-1 expression on clinical outcome in age-defined subgroups of patients with classical Hodgkin lymphoma. Blood.

[11] Jarrett  RF, Stark  GL, White  J (2005). Impact of tumor Epstein-Barr virus status on presenting features and outcome in age-defined subgroups of patients with classic Hodgkin lymphoma: a population-based study. Blood.

[12] Claviez , Tiemann , Luders  (2005). Impact of latent Epstein-Barr virus infection on outcome in children and adolescents with HL. J Clin Oncol.

[13] Twu  CW, Wang  WY, Liang  WM (2007). Comparison of the prognostic impact of serum anti-EBV antibody and plasma EBV DNA assays in nasopharyngeal carcinoma. Int J Radiat Oncol Biol Phys.

[14] Ishii  H, Takahara  M, Nagato  T (2012). Monocytes enhance cell proliferation and LMP1 expression of nasal natural killer/T-cell lymphoma cells by cell contact-dependent interaction through membrane-bound IL-15. Int J Cancer.

[15] Utsuki  S, Oka  H, Miyajima  Y (2011). Epstein-Barr virus (EBV)-associated primary central nervous system lymphoma: is incidence of EBV expression associated with median survival time?. Brain Tumor Pathol.

[16] Lima  RT, Seca  H, Brás  S (2011). Treatment of Akata EBV-positive cells with doxorubicin causes more EBV reactivation than treatment with etoposide. Chemotherapy.

[17] Machado  AS, Da Silva Robaina  MC, Magalhães De Rezende LM (2010). Circulating cell-free and Epstein-Barr virus DNA in pediatric B-non-Hodgkin lymphomas. Leuk Lymphoma.

